# The Modulation of Sucrose Nonfermenting 1-Related Protein Kinase 2.6 State by Persulfidation and Phosphorylation: Insights from Molecular Dynamics Simulations

**DOI:** 10.3390/ijms241411512

**Published:** 2023-07-15

**Authors:** Miaomiao Li, Ting Wu, Shuhan Wang, Tianqi Duan, Siqi Huang, Yanjie Xie

**Affiliations:** 1 College of Life Sciences, Nanjing Agricultural University, Nanjing 210095, China2021116101@stu.njau.edu.cn (S.W.);; 2 Institute of Bast Fiber Crops (IBFC), Chinese Academy of Agricultural Sciences (CAAS), Changsha 410205, China

**Keywords:** hydrogen sulfide, persulfidation, phosphorylation, SnRK2.6, molecular dynamics simulation, ABA

## Abstract

SnRK2.6 (SUCROSE NONFERMENTING 1-RELATED PROTEIN KINASE2.6) has been characterized as a molecular switch for the intracellular abscisic acid (ABA) signal-transduction pathway. Normally, SnRK2.6 is kept in an “off” state, forming a binary complex with protein phosphatase type 2Cs (PP2Cs). Upon stressful conditions, SnRK2.6 turns into an “on” state by its release from PP2Cs and then phosphorylation at Ser175. However, how the ”on” and “off” states for SnRK2.6 are fine-tuned, thereby controlling the initiation and braking processes of ABA signaling, is still largely unclear. SnRK2.6 activity was tightly regulated through protein post-translational modifications (PTM), such as persulfidation and phosphorylation. Taking advantage of molecular dynamics simulations, our results showed that Cys131/137 persulfidation on SnRK2.6 induces destabilized binding and weakened interactions between SnRK2.6 and HAB1 (HYPERSENSITIVE TO ABA1), an important PP2C family protein. This unfavorable effect on the association of the SnRK2.6–HAB1 complex suggests that persulfidation functions are a positive regulator of ABA signaling initiation. In addition, Ser267 phosphorylation in persulfidated SnRK2.6 renders a stable physical association between SnRK2.6 and HAB1, a key characterization for SnRK2.6 inhibition. Rather than Ser175, HAB1 cannot dephosphorylate Ser267 in SnRK2.6, which implies that the retained phosphorylation status of Ser267 could ensure that the activated SnRK2.6 reforms the binary complex to cease ABA signaling. Taken together, our findings expand current knowledge concerning the regulation of persulfidation and phosphorylation on the state transition of SnRK2.6 and provide insights into the fine-tuned mechanism of ABA signaling.

## 1. Introduction

Under the circumstances of global warming, it appears that there is a higher prevalence of extreme climate events such as drought, flooding, salinity, and heat, all of which put plant growth and development at risk. To respond to the stresses caused by extreme climatological changes, plants have evolved certain effective regulatory mechanisms to ensure their survival rate. Post-translational modifications (PTMs) act as the main mechanism among these mechanisms. Plant tolerance to these abiotic stresses or their combinations might be adjusted by one PTM or interactions between PTMs. Phosphorylation, glycosylation, and acetylation are common PTMs that modify protein activity, and some other PTMs documented relatively later also have a regulating role in plant response [1].

Some important signal molecules are reported to exert their regulatory roles through PTMs, such as hydrogen sulfide (H_2_S). Protein persulfidation has been well documented, which may be the main pathway by which H_2_S acts as a signaling molecule in organisms [2]. Persulfidation occurs on the cysteine residue (C) of target proteins, which converses from the C-SH group to the C-SSH group [3]. Although H_2_S cannot react with thiol, its reaction with oxidized cysteine residues is possible [4]. The oxidized forms include sulfenic acid (–SOH), nitrosothiuls (–SNO), and some others, in which sulfenic acid reacting with H_2_S is the most thermodynamically favorable [5,6]. In particular, since a larger amount of persulfidation protein in *Arabidopsis thaliana* [7] was identified based on the persulfidation proteome data, there were a considerable number of reports about the regulation of plant life activities by persulfidation [8,9,10,11]. As a consequence of this PTM, the target protein undergoes conformational or charge changes, leading to altered function [12]. Proteins involved in various biological pathways are persulfidated, which could alter enzymatic activity, binding capacity, intracellular location, etc. [7,13,14,15,16]. For example, persulfidation at C44 and C205 in DES1 (L-CYSTEINE DESULFHYDRASE 1), an important H_2_S-producing enzyme, elevates its activity and concomitant H_2_S production and then amplifies the H_2_S signal in Arabidopsis [17].

Plant physiological response is largely coordinated by phytohormones, whose critical role is beyond question under adverse conditions [18]. Abscisic acid (ABA) synthesis is one of the fastest responses to plant stresses [19]. ABA was recognized as a “plant stress resistance factor” [20], especially involved in regulating stomatal closure, and reported to control the expression of many stress-responsive genes in plants [21,22,23]. Stomatal movement regulates the exchange of O_2_ and CO_2_ with the environment and transpiration-associated water loss. ABA promotes stomatal closure and inhibits stomatal opening, allowing plants to retain water under drought stress, one of the main causes of global crop losses [24]. A central signaling complex is responsible for ABA signal perception [25]. This complex consists of PYRABACTIN RESISTANCE/PYR-LIKE/REGULATORY COMPONENT OF ABA RECEPTOR (PYR/PYL/RCAR) as ABA receptors, protein phosphatase type 2Cs (PP2Cs) as negative regulators of ABA signaling [26], and Sucrose Nonfermenting 1-RELATED PROTEIN KINASE2 (SnRK2s) as a major switch for downstream signaling [27]. It was proposed that under normal growth conditions with low cellular ABA levels, PP2Cs bind to SnRK2s to keep the SnRK2s in an inactive state. However, when plants are subjected to stress conditions, elevated cellular ABA binds PYR/PYL/RCARs, which in turn bind PP2Cs, resulting in the release of SnRK2s from PP2Cs. Subsequently, SnRK2s self-activated via auto-phosphorylation to trigger various ABA-induced physiological responses [25,28].

**Figure 1:**
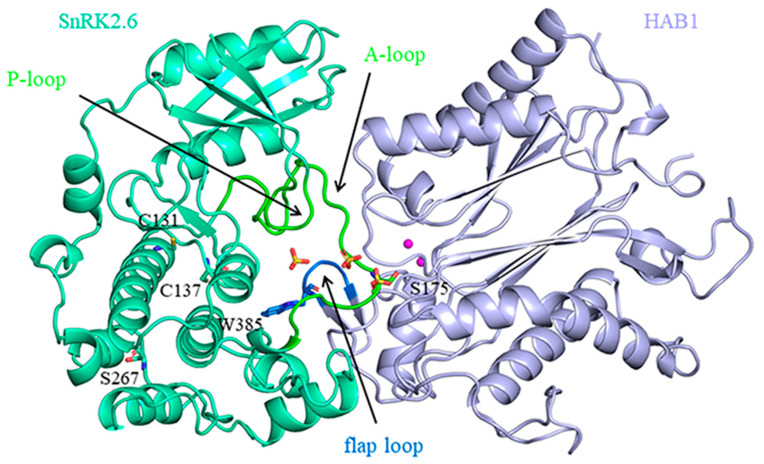
Structural information of the SnRK2.6–HAB1 complex (PDB code: 3UJG). The SnRK2.6 and HAB1 proteins are shown as cartoons. The important residues (S175 in SnRK2.6 and W385 in HAB1) and the modified residues (C131, C137, and S267 in SnRK2.6) are shown as sticks. The Mg^2+^ ions are shown as magenta spheres, and the SO4^2−^ ions are shown as sticks.

In recent years, persulfidation modification has been shown to play a key role in ABA-induced stomata closure [16]. SnRK2.6, also known as open stomata (OST1), belongs to the subclass III SnRK2s and is a serine (S)/threonine (T) protein kinase activated by phosphorylation [29]. As a central component of the ABA signaling network, the presence of persulfidated residues in SnRK2.6 was indicated according to the persulfidation proteome data [30]. Chen et al. [24] validated that persulfidation occurred in SnRK2.6 at residues C131 and C137, which promoted its activity and positively regulated ABA signaling. They also demonstrated that persulfidation-induced SnRK2.6 activity was regulated by the phosphorylation status of S175 and S267 [31]. HAB1, the best-characterized PP2C in ABA signaling [32], binds to and dephosphorylates SnRK2s, especially SnRK2.6, and negatively regulates ABA signaling [33,34]. In the structure of the SnRK2.6–HAB1 complex (Figure 1), the interface shows a striking similarity with the receptor-PP2C interface, manifested by the SnRK2.6 kinase activation loop (A-loop) docking into the active site of PP2C. Additionally, the conserved ABA-sensing tryptophan of PP2C inserts into the SnRK2.6 kinase catalytic cleft, thus mimicking receptor-PP2C interactions [35]. It is worth mentioning that HAB1 displays selective SnRK2.6 activation loop dephosphorylation. Hence, HAB1 could efficiently remove the phosphate group from S175 located at the A-loop but not from other phosphorylated residues in SnRK2.6, such as S267 [36]. Although experiment data provide detailed information about the regulatory role of persulfidation and phosphorylation on SnRK2.6 activity, the information on the two PTMs′ modulation of the SnRK2.6 state, which is closely related to the initiation and brake of ABA signaling has not been much explored.

In this study, we applied molecular dynamics (MD) simulations to shed light on modulating the state of the SnRK2.6 protein by persulfidation and phosphorylation. Four systems were considered, comprising a wild type and three modified types: persulfidated C137, persulfidated both C131 and C137, and the combination of persulfidated C131/C137 and phosphorylated S267. The simulations showed reduced SnRK2.6–HAB1 stabilization and weakened SnRK2.6–HAB1 interactions upon persulfidation of SnRK2.6. The induced effects of persulfidation establish a favorable molecular basis for dissociating SnRK2.6 from HAB1. Moreover, our study found that the co-existence of C131/C137 persulfidation and S267 phosphorylation on SnRK2.6 induced a rather stable SnRK2.6–HAB1 complex. This suggests that when persulfidated SnRK2.6 switched back to the off state, the phosphorylation status of Ser267 rendered an intermediate state of the SnRK2.6–HAB1 complex to ensure the brake of ABA signaling. On account of the SnRK2.6 kinase protein serving as a key regulation protein related to drought in plants, our findings expand current knowledge about the regulatory mechanism of PTMs on SnRK2.6. This provides a comprehensive understanding of SnRK2.6 regulation, which is of great scientific significance for improving plant drought tolerance.

## 2. Results

We carried out three replicate explicit-solvent MD simulations for the SnRK2.6–HAB1 heterodimer in four forms, denoted as WT, C137SSH, 2CSSH, and 2CSSH-pS267, respectively, in the next description. Firstly, the Cα root-mean-square deviations (RMSDs) of the SnRK2.6–HAB1 heterodimer were monitored during the simulations. As shown in Figure S1, most of the SnRK2.6–HAB1 complex was stable in the simulation process as the RMSD values were smaller than 4 Å. It was noted that the proteins almost reached a steady state in the last 200 ns. Therefore, most analyses were performed on the 200 ns equilibrated trajectories for all systems.

### 2.1. The Opposite Movement in SnRK2.6–HAB1 Heterodimer Induced by Persulfidation of SnRK2.6

**Figure 2:**
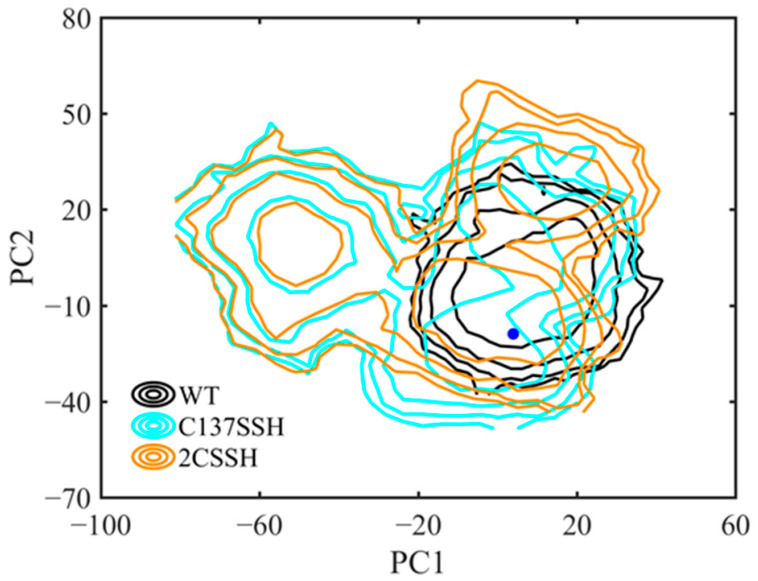
Conformational spaces of three systems of the first two PCs. PCA calculations were based on Cα atoms. The starting conformation of the SnRK2.6–HAB1 complex is shown as a blue dot.

The principal component analysis (PCA) was employed to characterize the essential dynamics of the simulated systems. The first two principal components contributed to the protein motion for 27.14% and 20.87%, respectively; others contributed less than 10%. Thus, we focused on the first two prominent components. To compare the conformational sampling for the SnRK2.6–HAB1 complex in wild-type and persulfidated forms, the distributions of conformations in the plane of the first two principal components (termed PC1 and PC2) were translated to free energy surfaces according to the Boltzmann relation. The breadth of these surfaces suggests the flexibility of the system. The PCA results exhibited the distinct conformational distributions of the three systems. As displayed in Figure 2, the C137SSH and 2CSSH systems covered larger overlaps in conformational space and sampled a broader distribution with more than one free energy basin. Moreover, most of the conformational sampling deviated from the original conformation. However, the protein complex was confined to a certain conformational space with one free energy basin for the WT system. It was noted that the two more flexible systems were mainly distinguished from the WT one by PC1. The mode derived from the first principal component was relative to the opposite movement between SnRK2.6 and HAB1 (Figure S2A). Along the negative PC1 direction, this movement made the SnRK2.6 protein move away from the HAB1 protein. Hence, the persulfidation of SnRK2.6 induced dynamical and conformational perturbations of the SnRK2.6–HAB1 heterodimer.

**Figure 3:**
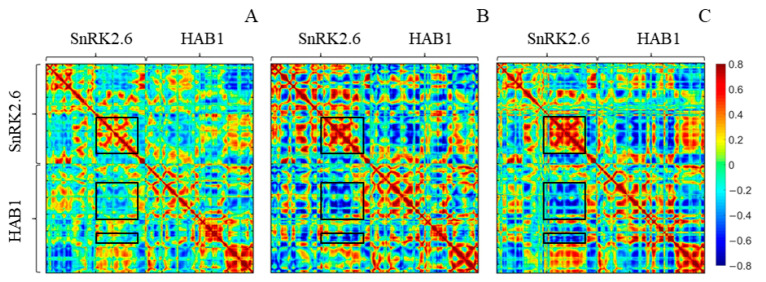
The dynamic cross-correlation matrix map of SnRK2.6–HAB1 complex in each system: (**A**) WT system; (**B**) C137SSH system; and (**C**) 2CSSH system. The C-lobe of the SnRK2.6 kinase domain is demonstrated by an upper black square box, and the lower helical structure region and loop region adjacent to the flap loop of HAB1 are indicated by lower boxes.

In addition, the dynamic cross-correlation matrices (DCCMs) for the heterodimer were calculated for more information. According to the DCCMs (Figure 3), for the C137SSH and 2CSSH systems, enhanced intermolecular correlations were observed, especially an intensified negative correlation between the C-lobe of the SnRK2.6 kinase domain and the lower helical structures as well as loop region adjacent to the flap loop in HAB1. These observations aligned with the opposite movement of two proteins obtained from PC1.

### 2.2. Persulfidation of SnRK2.6 Weakens Its Binding with HAB1

The affinity and specificity between two proteins are finely tuned according to their functions. Chen et al. [37] observed a direct relationship between buried interfacial surface area and affinity. In other words, as buried surface area increased, binding affinity increased.

**Figure 4:**
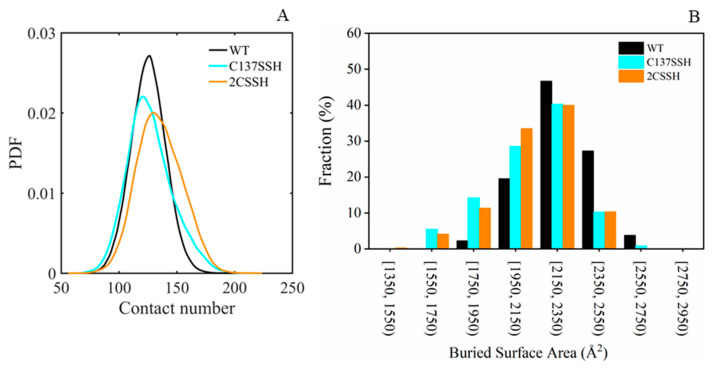
The assessment of SnRK2.6–HAB1 binding. (**A**) The probability distribution function (PDF) of atom–atom contact numbers between SnRK2.6 and HAB1 with heavy atoms considered. (**B**) Interface size distribution.

As the opposite movement between two proteins was observed in persulfidated systems, we then roughly assessed the binding of SnRK2.6 to HAB1 through the number calculation of atom–atom contact pairs and the buried interfacial surface area calculation. As seen in Figure 4A, although the contact number slightly increased in the C137SSH system and increased in the 2CSSH system more than in the WT system, a broader distribution was presented in the two persulfidated systems. This suggests the binding stability decreased between SnRK2.6 and HAB1 for the C137SSH and 2CSSH systems. As shown in Figure 4B, for the WT system and two persulfidated systems, about 40% of interfaces were in the 2150–2350 Å^2^ range. Moreover, the second size of interface is distributed in the 2350–2550 Å^2^ range for the WT system and the 1950–2150 Å^2^ range for the two persulfidated systems. These results illustrated that the cysteine(s) that underwent persulfidation indeed exerted influence on the association of the SnRK2.6–HAB1 complex.

**Figure 5:**
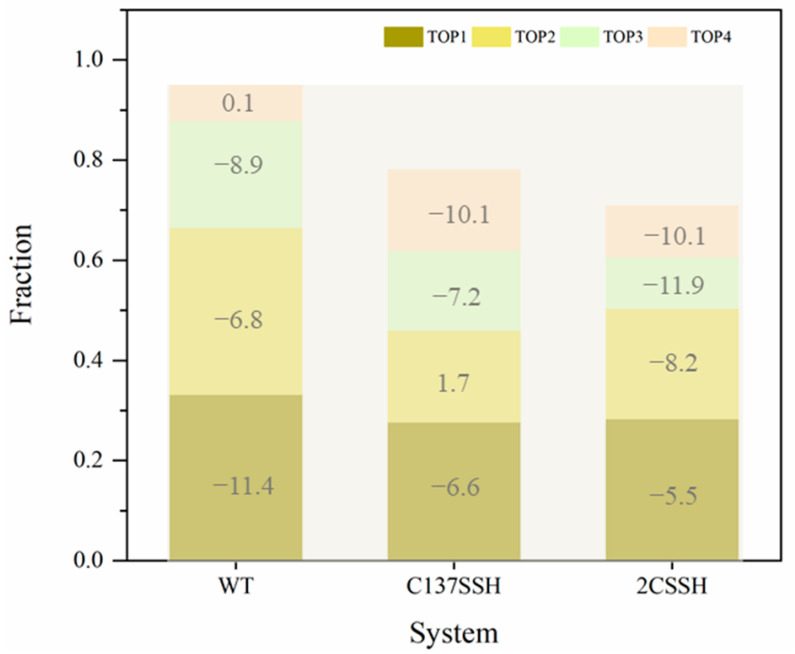
The clustering information of protein conformations in all systems. The top four clusters of the SnRK2.6–HAB1 complex and the Δ^i^G value of each cluster representative were measured in PISA software.

Subsequently, PISA software (https://www.ebi.ac.uk/msd-srv/prot_int/ (accessed on 23 November 2022)) [38,39] was used to calculate the chemical properties of the SnRK2.6–HAB1 interface, such as the free energy of dissociation. First, the Cα RMSD-based cluster analysis with a 1.35 Å distance cutoff was applied to the concatenated trajectories of three parallel simulations considering the equilibrated stage. It can be seen from Figure 5 that the proportion of the largest cluster was higher in the WT system compared to the C137SSH and 2CSSH systems. In addition, the sum of shares of the first four clusters was 0.95 in the WT system, 0.78 in the C137SSH one, and 0.71 in the 2CSSH one, respectively, which further demonstrates the increased conformational flexibility of the SnRK2.6–HAB1 complex upon persulfidation. Then, the four representative conformations of each system were taken as input into the PISA software. The negative Δ^i^G (kcal/mol) value corresponded to the hydrophobic interface or positive affinity, while the positive value corresponded to the opposite. As for the largest cluster, the representative conformation gained a q value of −11.4 for the WT system, −6.6 for the C137SSH one, and −5.5 for the 2CSSH one. Taken together, the above results suggest that persulfidation negatively regulates the SnRK2.6–HAB1 association, which might contribute to the dissociation between them on a larger timescale beyond our simulation time.

### 2.3. The Phosphorylation Status of S267 Affects the Stability of SnRK2.6–HAB1 Complex

**Figure 6:**
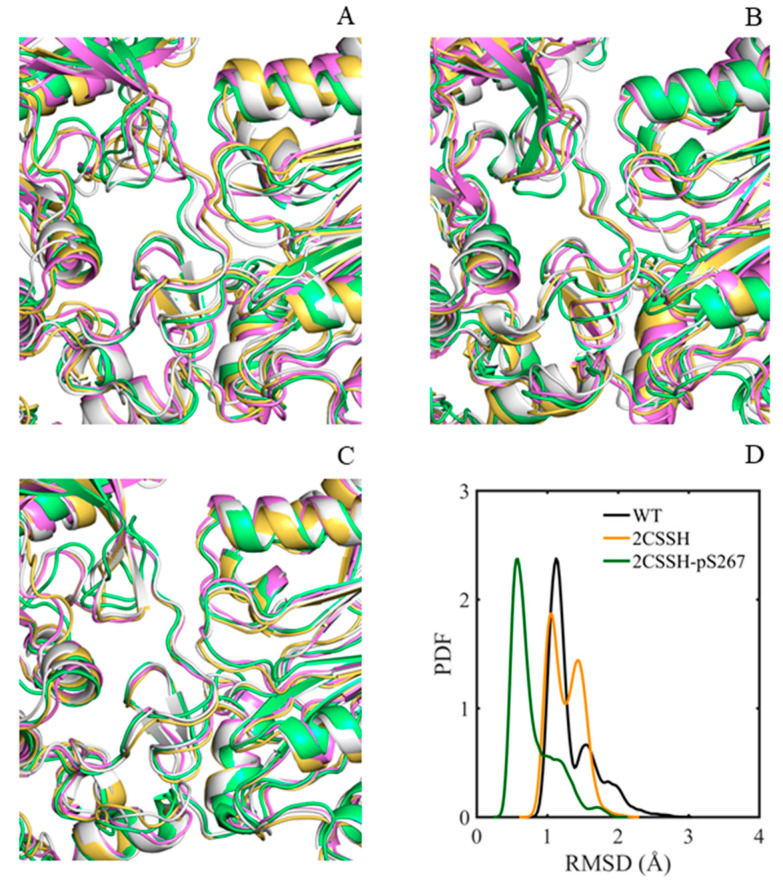
Superposition of the top four representative conformations obtained by clustering the concatenated equilibrated trajectories. The top four cluster representative structures are colored white, lime green, violet, and yellow–orange, respectively. (**A**) WT system; (**B**) C137SSH system; (**C**) 2CSSH-pS267 system. (**D**) The probability distribution profile of the RMSD values of the three loop residues. The RMSDs were calculated according to their average coordinates based on equilibrated trajectories.

As mentioned above, persulfidation modification plays a positive role in free state formation for SnRK2.6. However, the activity of SnRK2.6 is not needed for a long time to ensure plant growth. Thus, it has to be in the off state upon stress response, achieved by its dephosphorylation and physical interaction with HAB1. The inability of HAB1 to remove phosphorylation outside of the SnRK2.6 activation loop should also be mentioned. According to the status of C131/C137 and the status of other phosphorylation sites, several intermediate states of the SnRK2.6–HAB1 complex might exist during the brake process of ABA signaling. We next focused on the three systems (WT, 2CSSH, and 2CSSH-pS267). By aligning the representative conformations within systems, major differences were observed in the P-loop and the A-loop of SnRK2.6 protein and a minor discrepancy in the flap loop of HAB1 protein, besides the exterior secondary structures’ exposure to solvent (Figure 6A–C).

Given the three loops located at the SnRK2.6–HAB1 interface, whose flexibilities directly regulate protein binding, we further performed the dynamics analyses for the three interfacial loops. The Ca RMSDs of residues in the three loops were calculated according to their average coordinates based on equilibrated trajectories. As illustrated in Figure 6D, for the 2CSSH-pS267 system, although some values were greater than 1 Å, most RMSD values were less than 1 Å. Thus, the loops were relatively stable during the simulations. For the other two systems, the average RMSD value was greater than 1 Å and less than 1.5 Å, especially for the 2CSSH system, where a bimodal distribution of RMSD was observed. Therefore, the loops were more dynamic than in the 2CSSH-pS267 system on the whole. Moreover, the root mean square (RMS) fluctuations of the first three eigenmodes for the loops were calculated from a mass-weighted covariance matrix relying on PCA. The respective motility of the three loops differed, and the most flexible section varied (Figure S3). To be more specific, for the WT system, the P-loop and the A-loop (S166–K174) were characterized by obvious motions and slight motions for the flap loop. For the 2CSSH system, the P-loop and the A-loop (H170–V177) were more mobile. Additionally, for the 2CSSH-pS267 system, the three loops appeared to have less movement than the A-loop residues (S164, K165, and S166). The results suggested an almost quenching of us dynamics for the 2CSSH-pS267 system, characterized by decreased flexibility of the SnRK2.6–HAB1 complex and relative rigidification of the SnRK2.6–HAB1 interface.

### 2.4. A Quite Stable Intermediate State of SnRK2.6–HAB1 Complex

**Figure 7:**
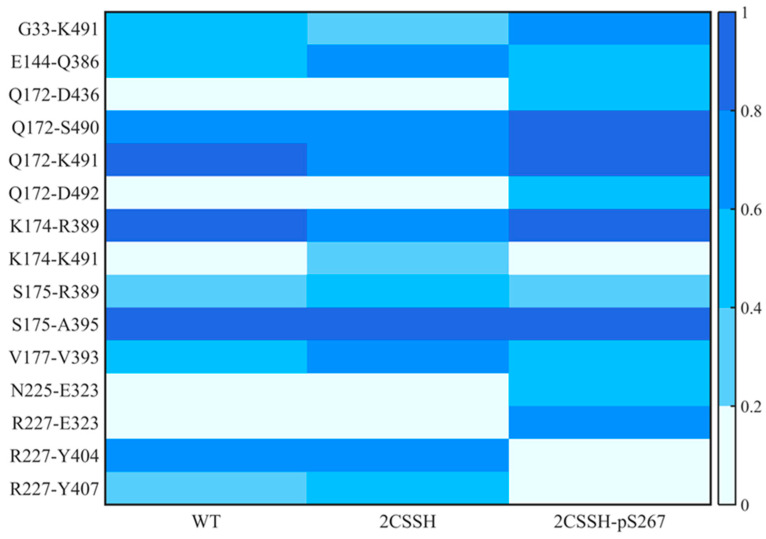
The hydrogen-bond interactions formed between SnRK2.6 and HAB1. The color indicates the proportion of a hydrogen bond during the simulation.

The prominent feature of the complex is the mutual packing of the kinase domain of SnRK2.6 and the phosphatase activation site of HAB1. Three separate regions in the SnRK2.6 kinase domain directly participate in binding to HAB1, including the A-loop, the catalytic cleft, and the αG helix. Polar and non-polar interactions drive the SnRK2.6–HAB1 association (Figure S4). The previous dynamical and conformational analyses underlined the dynamic change in the SnRK2.6–HAB1 interface. Therefore, we next compared variations of the extensive interactions between SnRK2.6 and HAB1 in the three systems following the two metrics. For a better description, the hydrogen bonding formed by residues prior to site 175 on the sequence was termed the upper region hydrogen bonding from a spatial perspective, otherwise the lower region hydrogen bonding. As shown in Figure 7, the upper region hydrogen bonding attenuated slightly in the WT system, weakened in the 2CSSH system, and was well maintained in the 2CSSH-pS267 system. For the lower region hydrogen bonding in the crystal structure of SnRK2.6–HAB1, the interaction involved in residue R227 still existed in the 2CSSH-pS267 system. Still, it was aborted in the other two systems, where new interactions formed.

**Figure 8:**
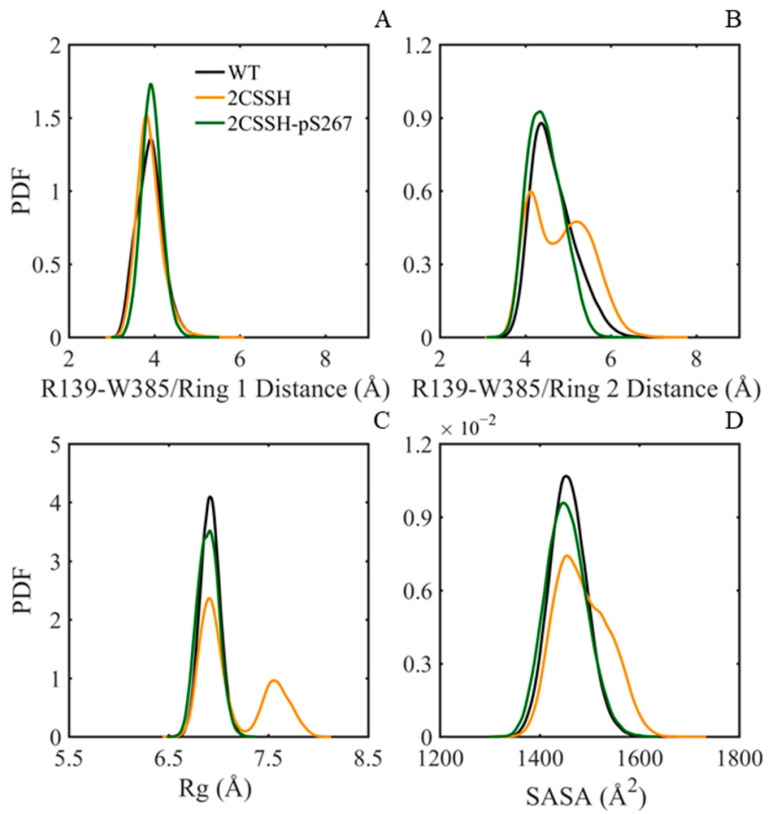
The intermolecular interaction information, including cation-π interaction and hydrophobic interaction. The probability distribution profile of the distance between the atom NZ of R139 in SnRK2.6 and the six-membered ring of W385 (**A**) and the five-membered ring of W385 (**B**) in HAB1. For residue W385, the center of mass of the sidechain ring was selected as the distance calculation object. The probability distribution profile of the *R*_g_ value (**C**) and solvent-accessibility surface area value (**D**) of hydrophobic residues.

In addition, the flap loop in HAB1 was inserted deeply into the SnRK2.6 kinase catalytic cleft, which induced cation-pi interaction formation between R139 in the catalytic loop and W385 in the flap loop. The cation-π interaction and the more conventional interactions together make a significant contribution to the overall stability of most proteins [40]. To characterize the interaction, the distance between the sidechains of R139 and W385 was calculated (Figure 8A,B). The average value of the distance between R139 and the six-membered ring of W385 was no more than 4 Å, and the one between R139 and the five-membered ring of W385 was no more than 5 Å for the three systems. This suggests that the cation-π interactions were well maintained during the simulations, reflecting that this interaction played an important role in the SnRK2.6–HAB1 association. According to the two measured distance distributions, it could be found that the cation-π interaction was most stable in the 2CSSH-pS267 system and had relatively less stability in the 2CSSH system.

In addition, some hydrophobic residues are located in the binding region consisting of the SnRK2.6 αG helix and the HAB1 loop region adjacent to the flap loop. Then, we evaluated the hydrophobic core through R_g_ and solvent-accessible surface area (SASA) calculations. As presented in Figure 8C,D, the hydrophobic core was more stable in the WT system with an average R_g_ value of 6.92 ± 0.10 Å and an average SASA value of 1457.8 ± 37.34 Å^2^, as well as in the 2CSSH-pS267 system with an average R_g_ value of 6.90 ± 0.11 Å and an average SASA value of 1453.6 ± 41.06 Å^2^. In contrast, the hydrophobic core in the 2CSSH system was relatively flexible, characterized by an average R_g_ value of 7.14 ± 0.34 Å and an average SASA value of 1485.7 ± 52.59 Å^2^. A brief summary of the interaction comparisons among the three systems showed that the interactions contributing to the binding of the SnRK2.6–HAB1 complex appeared to have better maintenance in the 2CSSH-pS267 system than in the 2CSSH system. Based on the dynamical analysis and interaction comparison results, a stable SnRK2.6–HAB1 complex could be revealed, in which C131/C137-associated persulfidation together with S267-associated phosphorylation occurred on SnRK2. This indicates that the status of S267 may have a regulatory role in the SnRK2.6 state.

### 2.5. Calculated S^2^ Order Parameter Identifies Three Key Residues Responsible for SnRK2.6–HAB1 Interface Disturbance

**Figure 9:**
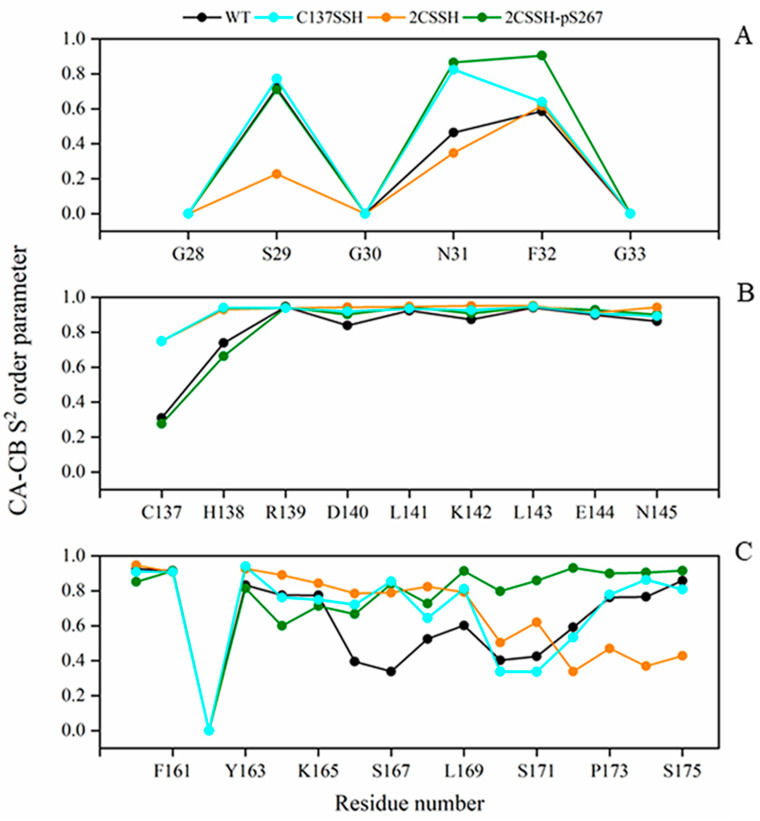
The S^2^ order parameter for Cα–Cβ vectors of residues in the P-loop (**A**), the catalytic loop (**B**), and the A-loop (**C**).

Our foregoing analyses revealed a distinct effect on SnRK2.6–HAB1 binding upon persulfidation modification of SnRK2.6 in the presence and absence of phosphorylation. To determine the determinant behind the discrepancy, more detailed information is necessary. Therefore, we focus on residue level in the next analyses, considering the four simulated systems. The generalized order parameter, S^2^, is an appropriate indicator of protein backbone motions on computationally feasible timescales [41]. We applied the S^2^ order parameter for the Cα–Cβ vector to estimate residue flexibility in several important structures (the P-loop, the catalytic loop, and the A-loop in SnRK2.6). As seen in Figure 9, for the P-loop, residue F32 exhibited lower-order parameter values in the other three systems except for the 2CSSH-pS267 system. For the catalytic loop, residue C137 showed a decreased order parameter relative to other residues in this loop in all systems. Additionally, residues Y153–K165 presented the lowest order parameter for the A-loop in the 2CSSH-pS267 system. According to the above observations, several residues (F32, Y163, and K165) were not close to C137 in sequence, so we hypothesized that the three residues might be crucial for enlarging the mobility of C137.

**Figure 10:**
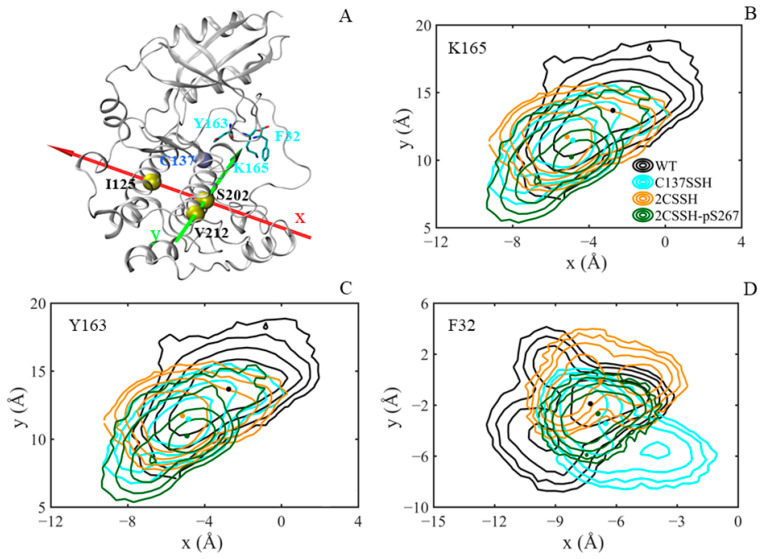
The displacement of the sidechain of three residues (K165, Y163, and F32). (**A**) The defined coordinate system: Cα of S202 in αF helix is at the origin, the x-axis goes through Cα of I125 in αE helix, and the y-axis goes through Cα of V212 in αF helix. The calculated residues are shown in cyan sticks, and the Cα atom of C137 is shown in ice-blue spheres. (**B**) Corresponding results for the center of K165 sidechain heavy atoms in the x–y plane. (**C**) Corresponding results for the center of Y163 sidechain heavy atoms in the x–y plane. (**D**) Corresponding results for the center of F32 sidechain heavy atoms in the x–y plane. The average position of the three residues in each system is shown as a dot with a matching color.

To confirm this, the movement of the sidechains of the three residues was considered and characterized by virtue of the defined coordinated system (Figure 10). Through observing the structure of the SnRK2.6–HAB1 complex, it is found that the Y163 sidechain is very close to the P-loop, and the sidechain of F32, a residue in the P-loop, is close to the A-loop (H170–S175). For the residue K165, the four average positions shown as dots with matching colors suggested different directions for K165 motion in all systems. Specifically, the position of K165 on the x-axis was comparable in three modified systems and was maximal in the WT system. And the position of K165 on the y-axis was minimal in the 2CSSH-pS267 system, comparable in the C137SSH and 2CSSH systems, and maximal in the WT system. Due to the movement of K165, the N terminal of the A-loop (D160–S167) was shifted relative to the initial position, resulting in the positional change in Y163. Based on spatial structure, the displacement of the Y163 sidechain could induce the conformational flexibility of the P-loop, and the dynamic signal was then transmitted to the A-loop by the movement of F32. For the residue Y163, the average position of each system was individually dispersed, which might result in conformational heterogeneity of the P-loop in four systems. For the residue F32, this sidechain sampled the least space of positions in the 2CSSH-pS267 system, exerting less perturbation on the A-loop, consistent with the RMS fluctuation analysis.

### 2.6. The Inward-Facing Conformation of K165 Sidechain Underlines the Stable SnRK2.6–HAB1 Interface

In the crystal structure of the SnRK2.6–HAB1 complex, there were three sulfate ions, one located at the catalytic cleft region of the SnRK2.6 protein (Figure S5). Analyzing the surrounding environment, it was found that there were several polar and positive residues. Therefore, the existence of the sulfate ion might be to form coordinated interactions with the protein residues to make the SnRK2.6–HAB1 complex stable. Then, the distance between residue K142 and the sulfate ion was monitored during the whole simulation to observe the stability of the sulfate ion at the binding site. As shown in Figure S6, the sulfate ion was stable during the simulations performed for the 2CSSH-pS267 system, as there was no significant distance variation between residues K142 and SO4^2−^. However, in the other three systems, the divalent negative ion was away from the binding site observed during the two simulations performed. With the departure of the coordinated sulfate ion, there would be changes in the surrounding protein environment and possible increased motility of some residues, even causing disturbances in protein–protein binding.

The distance calculation was additionally performed on the sulfate ion with residue K165. Although the K165 sidechain points into the solvent in the starting structure, coordinated interaction formation is possible because of multiple rotation points in the lysine sidechain. According to the distance variations, K165 did coordinate with the sulfate ion in the 2CSSH-pS267 system since the distance between the two was mostly at 3 Å during the simulations (Figure S7). The coordination formation reflected a sidechain change in K165 from an outward-facing conformation at the start to an inward-facing one. This also explained the previous observation that the average K165 sidechain position was minimal on the y-axis in the 2CSSH-pS267 system. Hence, these results emphasized K165 as a key residue for the SnRK2.6–HAB1 association. The motility of K165 could be attributed to two aspects: the multiple rotation points of its sidechain and the induction by other residues through the coupling of residues. There are hydrogen bonds between the main chains of C137 and D140 and between the main chain of D140 and the sidechain of K165.

## 3. Discussion

As central components of ABA signaling, the conformational states of three key proteins (PYR/PYL/RCAR, PP2Cs, and SnRK2s) are crucial to transmitting ABA signals. Intracellular ABA concentration may govern the equilibrium among PYR/PYLs, PP2Cs, and SnRK2s. Under normal ABA levels, a kinase-phosphatase binary complex is present. However, when ABA concentration is high, an ABA-receptor-phosphatase ternary complex is formed [28]. Hence, the equilibrium between the free and complex states of SnRK2s fine-tunes ABA signaling, and the release of SnRK2s from PP2Cs resulting in a free form serves as a prerequisite for relaying the ABA signal.

**Figure 11:**
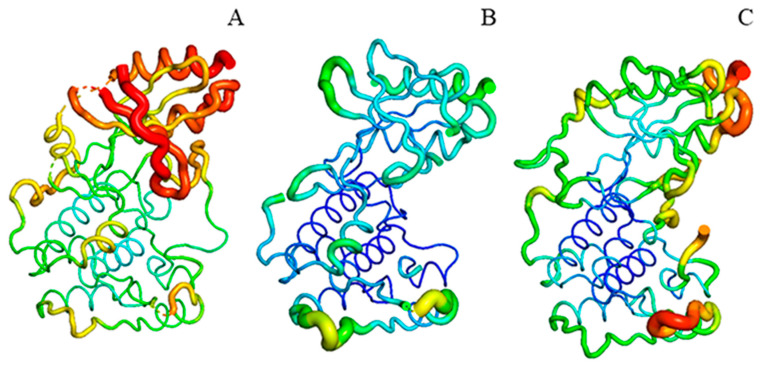
The mapping of b-factor values into crystal structures. (**A**) Cartoon representation of SnRK2.6 associated with HAB1 (PDB codes: 3UJG). For better comparison, the HAB1 protein is not shown. (**B**,**C**) Cartoon representation of SnRK2.6 in free form. SnRK2.6 (PDB code: 3UC3 [42]) (**B**) and SnRK2.6 (PDB code: 3UC4 [42]) (**C**).

Comparing the structures of the SnRK2.6 protein in free and complex states, it was found that the C-lobe of the kinase domain largely overlapped in two forms. At the same time, there were several differences between the two states in the N-lobe, including the P-loop, the αC-helix, and the A-loop. We then mapped the b-factor values recorded in the PDB file into respective structures, in which the b-factor metric reflects the conformational state of the protein in the crystal (Figure 11). The higher the value, the more unstable the conformation of the corresponding site. It was found that for SnRK2.6 in free form, most residues of the A-loop were missing, suggesting high conformational flexibility of the structural element. The A-loop was completed for the one complexed with HAB1, with only a few N-terminal residues exhibiting higher flexibility. Furthermore, the P-loop and the αC-helix appeared to significantly enhance mobility for SnRK2.6 in complex form than in free form, seemingly as compensation for the diminished flexibility of the A-loop in a complex state. Therefore, the conformational dynamics of the P-loop might be critical for the state transition of SnRK2.6.

**Figure 12:**
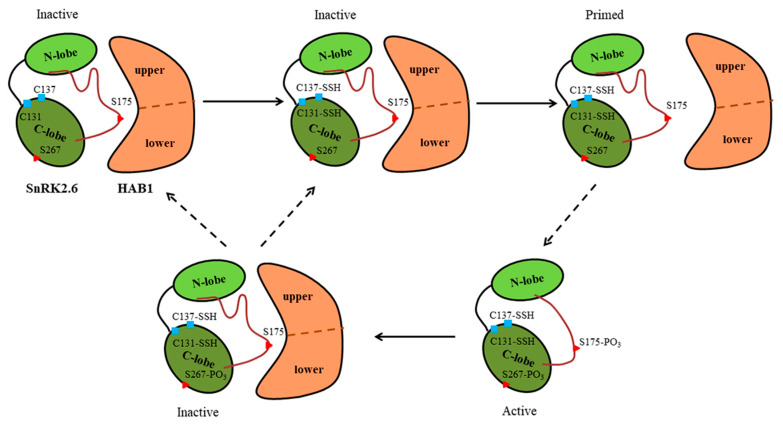
A model for persulfidation and phosphorylation regulation of the SnRK2.6 state and thus controlling ABA signaling. The activation loop (A-loop) in SnRK2.6 is colored dark red. Red triangles denote phosphorylated sites, and blue squares denote persulfidated sites. The dotted lines represent hypothetical regulations. Under normal growth conditions, SnRK2.6 kinase forms a binary complex with HAB1 phosphatase. Under oxidative stress, there might be oxidation of cysteine residues in SnRK2.6. Upon persulfidation of the SnRK2.6, the SnRK2.6–HAB1 complex appears to have less stabilization and weakened interaction, which is adverse to heterodimerization between them. SnRK2.6 kinase released from HAB1 could be activated by B3 RAFs phosphorylating S175 in the A-loop. The activated SnRK2.6 might intermolecularly trans-phosphorylate other SnRK2.6, rending the phosphorylation of S267, a residue not in the A-loop. Under the interplay between persulfidation and phosphorylation, SnRK2.6 activity is further amplified, promoting ABA signaling. When ABA signaling is turned off, SnRK2.6 forms a binary complex with HAB1 again. HAB1 selectively dephosphorylates residue S175 but not S267. The C131/C137-persulfidated and S267-phosphorylated SnRK2.6 forms a more stable complex with HAB1. Therefore, it might be several undetermined phosphatases that remove phosphorylation sites outside of the A-loop of SnRK2.6 before the enablement of ABA signaling.

Through MD simulations of the SnRK2.6–HAB1 complex, we found that C131/C137-associated persulfidation induced conformational and dynamical effects. First, the persulfidation-induced opposite movement may damage the interaction interface. Second, persulfidation brought about conformational heterogeneity in the P-loop and a concomitant conformational change in F32. This could ultimately disturb the middle segment of the A-loop, which was directly participating in SnRK2.6–HAB1 binding. The two aspects indicate that persulfidation modification plays a positive role in the state transition of SnRK2.6, manifested by reducing the stability of the SnRK2.6–HAB1 complex and weakening the binding between the two proteins. This is conducive to the dissociation between them, establishing a favorable molecular basis for SnRK2.6 activation closely related to the initiation of ABA signaling. Upon SnRK2.6 being released from HAB1, it could be activated through phosphorylation. Chen et al. found that persulfidation on C131, C137, or both residues in SnRK2.6 could enhance the kinase activity, and the phosphorylation status of S267 further positively regulated the persulfidation level, contributing to ABA signaling [24,31]. Accordingly, the intramolecular interaction between phosphorylation and persulfidation participated in SnRK2.6 activation during ABA signaling. When ABA signaling is turned off, the status of several critical residues might exert a distinct effect on the inhibition of SnRK2.6 by HAB1, especially in the situation where S267 phosphorylation and C131/C137 persulfidation co-exist on SnRK2.6. In this state, the SnRK2.6–HAB1 complex appears more stable, as our study found. For the 2CSSH-pS267 system, less mobility is exhibited by the P-loop, less position space is sampled by F32, and the SnRK2.6–HAB1 interface is more rigid. Comparing our study with Chen et al.’s [31], it was found that the effect induced by C131/C137-associated persulfidation and S267-associated phosphorylation in the two studies exhibits the opposite. The conformational state of SnRK2.6 protein is a fundamental difference between our study and Chen et al.’s, in which SnRK2.6 complexed with HAB1 in the present study and was released from HAB1 in Chen et al.’s experiments. Therefore, it was suggested that the status of S267 might serve as a regulatory element in ABA signaling. Concretely, when SnRK2.6 activity is not needed, the phosphorylated status of residue S267 could well maintain the physical interaction between SnRK2.6 and HAB1, ensuring the inhibition of SnRK2.6. When one is needed, the phosphorylated status of S267 and persulfidated status of C131/C137 amplify the kinase activity of SnRK2.6 in synergy, facilitating ABA signaling. Finally, a model for persulfidation and phosphorylation regulation of the SnRK2.6 state was proposed, controlling ABA signaling (Figure 12). This model may provide a new perspective on SnRK2.6 regulation.

PTMs have emerged as major mechanisms for kinase regulation, mostly functioning through the induction of structural changes in the target protein. Several previous studies reported that the change in protein function resulted from the structural change induced by PTMs [43,44]. Indeed, Chen et al. confirmed that persulfidation-induced structural change established the positive correlation between phosphorylation and persulfidation, in which the structural change manifested by bringing the S175 residue in the A-loop close to the ATP-γ-phosphate proton acceptor site D140 and the Mg^2+^-binding site D160, thereby improving the transfer efficiency of γ-phosphate group [31]. In the present work, we found that the distinct sidechain conformation of the K165 residue in the A-loop underlined the quenching of us dynamics in the 2CSSH-pS267 system. For the 2CSSH-pS267 system, the conformation featuring the sidechain inward of K165 was more sampled, which made it possible to form coordination interactions with the interfacial SO4^2−^ ion, thereby ensuring the stabilization of the SnRK2.6–HAB1 heterodimer. The stable binding of the SnRK2.6–HAB1 complex emphasizes the HAB1 inhibition of SnRK2.6. The comparison of our work with Chen et al.’s work stresses that distinct structural changes induced by PTM(s) on target proteins may lead to various regulatory effects. Overall, the interplay of PTMs is complicated, and the generated effects on proteins, including stability and activity, may be either positive or negative. These effects depend on the redistribution of protein conformational states upon PTMs and whether they favor conformational states vital for the preferred functionality.

## 4. Materials and Methods

### 4.1. System Setup

There are four systems in the present study, including one wild type and three modified types, including persulfidation and phosphorylation modifications. The initial crystal structure of the SnRK2.6–HAB1 heterodimer was built based on the one downloaded from the Protein Data Bank (PDB code: 3UJG [35]). The two mutated residues in the crystal structure were replaced back by PYMOL [45] to obtain the wild-type SnRK2.6–HAB1 structure. The missing loop residues were added using the ProPrep module of Schrödinger Maestro [46]. Then, the other three structures were obtained by modifying the targeted residues of SnRK2.6 protein based on the structure of wild-type SnRK2.6–HAB1.

For all systems, the AMBER force fields ff14SB [47] were applied to proteins. Force field parameters for phosphorylated residue were acquired from the AMBER Parameter Database [48], and the ones for persulfidated residue were obtained by user-defined combining Gaussian [49], Multiwfn [50], and Antechamber [51] tools. The two most typical secondary structures of proteins are the alpha-helical and the beta-strand. From the perspective of the residues constituting them, the phi and psi angles of the residue backbone are different. Therefore, both conformations are taken into account when fitting the electrostatic potential charge of persulfidated residue. The structure optimization was first performed with the B3LYP/6-311G** method, followed by the single point energy calculation to optimize the electron function at the B3LYP/def2tzvp level of theory. The atomic charges were generated based on the obtained electron functions employing the RESP module in Multiwfn [50]. The key parameters (bond and torsion) for atoms were acquired using the Antechamber [51] tool. For the ions resolved with the SnRK2.6–HAB1 heterodimer in the crystal structure, the force field parameters of the SO4^2−^ ion were generated by Sobtop [52], and a 12-6-4 LJ-type nonbonded model [53] was employed for Mg^2+^ ions. Each system was solvated in a cubic box filled with TIP4P-EW waters [54] with a 10 Å buffer zone and neutralized by adding appropriate numbers of Na^+^ or Cl^−^.

### 4.2. Molecular Dynamics (MD) Simulation

The whole system was first energy-minimized in four stages. A series of position restraints were applied (force constants of 5.0, 2.0, 1.0, and 0 kcal mol^−1^ Å^2^, respectively) to all heavy atoms, the backbone atoms, and Cα atoms in turn. Then, the systems were heated to 300 K at 250 ps with a restraint of force constant of 10 kcal mol^−1^ Å^2^ imposed on non-hydrogen atoms. Following these steps, 4.5 ns equilibration was carried out on each system. These steps include two stages at the canonical (NVT) ensemble with restraint imposed on non-hydrogen atoms (force constants of 1.0 and 0.5 kcal mol^−1^ Å^2^) and three stages at the isothermal-isobaric (NPT) ensemble with restraint imposed on the backbone atoms and Cα atoms (force constants of 0.5, 0.1 and 0.1 kcal mol^−1^ Å^2^). The final production run was performed without constraint for each system at the NPT ensemble with a 2-fs timestep. Each system performed three replicate simulations in AMBER 18 [55], in which a 10 Å distance cutoff was used for nonbonded interactions, the particle mesh Ewald (PME) summation method [56] was used to treat electrostatic interactions, and the SHAKE algorithm [57] was used to constrain bond vibrations involving hydrogen atoms. Each parallel simulation time was 1.3 us for the C137SSH system and 1.0 us for the other three systems.

### 4.3. Buried Surface Area Calculation

The surface area buried at a protein–protein interface was calculated as the sum of the solvent-accessible surface area of the monomers minus the solvent-accessible surface area of the complex, using a sphere of radius 1.4 Å as the probe. The size we described for the total calculated interface area was not divided by two as interface symmetry was not present for the SnRK2.6–HAB1 heterodimer.

### 4.4. Cluster Analysis and Calculation of PPI Interface Properties

RMSD-based clustering of combined trajectories was accomplished using the DBSCAN algorithm [58]. The proportion of each cluster was calculated, and the similar conformations in the trajectories of the SnRK2.6–HAB1 complex were divided into the same cluster after clustering. The first four representative structures extracted based on the cluster analysis in each system were employed to calculate the chemical property of the SnRK2.6–HAB1 interface in PISA software [38].

### 4.5. Calculation of S^2^ Order Parameters

The isotropic reorientational eigenmode dynamics (iRED) method [59] was used to calculate S^2^ order parameters for Cα–Cβ vectors of protein residues, excluding glycine with no Cα–Cβ bond. iRED relies on a principal component analysis of the isotropically averaged covariance matrix, which is first calculated using the coordinates of Cα atoms of proteins by RMSD fitting and then diagonalized to obtain the principal component eigenvectors.

### 4.6. Analysis of MD Trajectories

The CPPTRAJ module [60] of the AMBER 18 software was used for general analyses, such as RMSD calculations, PCA analysis, and correlation analysis.

The root-mean-square deviation (RMSD) calculation was performed on Cα atoms with the first frame as a reference structure using the whole production trajectories. The RMSD calculation was conducted separately for each replicate simulation in each simulated system to evaluate the structural stability.

The Principal Component Analysis (PCA) [61] was performed using Cα atom coordinates obtained by concatenating equilibrated trajectories from all WT and persulfidated systems. The trajectories were projected onto the PCs associated with the two largest eigenvalues (PC1 and PC2) to capture the essential motions of proteins.

The residue–residue correlation map was generated by calculating the dynamical cross-correlation (DCC) matrices using Cα atoms coordinates of snapshots extracted from the equilibrated trajectories, considering three parallel simulations in each system.

## 5. Conclusions

In the present study, we applied MD simulations to reveal the possible modulation mechanisms of persulfidation and phosphorylation on SnRK2.6 involved in initiating and braking ABA signaling.

Our results demonstrate that persulfidation modification alone on SnRK2.6 induces more dynamical behavior of the SnRK2.6–HAB1 complex and results in attenuated interface interactions. Additionally, the phosphorylation status of S267 in persulfidated SnRK2.6 renders the dynamics of the SnRK2.6–HAB1 complex almost quenched, ensuring an even more stable association between SnRK2.6 and HAB1.

Hence, the persulfidation of SnRK2.6 establishes a favorable molecular basis for releasing SnRK2.6 from HAB1, a prerequisite for initiating ABA signaling. When activated, SnRK2.6 returns to the “off” state with the action of HAB1, and the phosphorylated S267 residue assures the reformation of the binary complex, then brakes the ABA signaling.

Our work reveals that persulfidation and phosphorylation could regulate ABA signaling by affecting the association of the SnRK2.6–HAB1 complex and also illustrates that the status of the S267 residue may play a role in persulfidation modulation on SnRK2.6.

The presence of such other phosphatases targeting SnRK2.6 should be investigated in the future due to HAB1 exhibiting selective activation loop dephosphorylation.

## Data Availability

Not applicable.

## Supplementary Materials

The following supporting information can be downloaded at: https://www.mdpi.com/article/10.3390/ijms241411512/s1.
