# Supplementary material for: The Modulation of Sucrose Nonfermenting 1-Related Protein Kinase 2.6 State by Persulfidation and Phosphorylation: Insights from Molecular Dynamics Simulations
Source: Int J Mol Sci. 2023 Jul 15;24(14):11512. doi: 10.3390/ijms241411512 (PMC10380758; doi:10.3390/ijms241411512)
Supplement: Supplementary file 1 [file ijms-24-11512-s001.zip › ijms-2444735-supplementary.pdf]

The Modulation of Sucrose Nonfermenting 1-Related Protein Kinase 2.6 State by Persulfidation and Phosphorylation:

Insights from Molecular Dynamics Simulations

Miaomiao Li [1], Ting Wu [1], Shuhan Wang [1], Tianqi Duan [1], Siqi Huang[2,] *, Yanjie Xie [1, 2,] *

1 College of Life Sciences, Nanjing Agricultural University, Nanjing 210095, China; 2021116101@stu.njau.edu.cn (S.W.)

2 Institute of Bast Fiber Crops (IBFC), Chinese Academy of Agricultural Sciences (CAAS), Changsha 410205, China

* Correspondence: huangsiqi@caas.cn (S.H.); yjxie@njau.edu.cn (Y.X.)

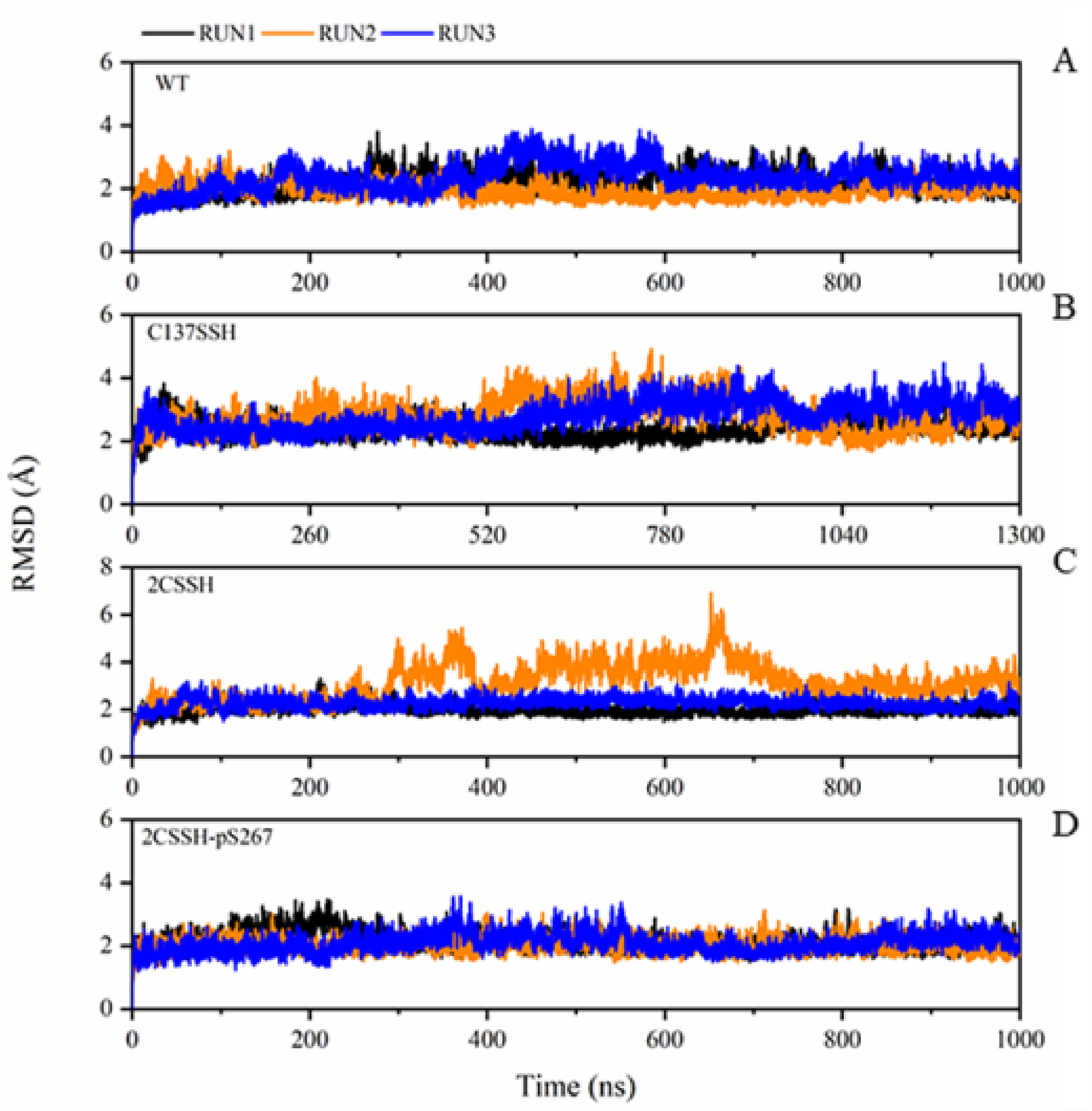

Figure S1. The RMSDs of SnRK2.6–HAB1 complex in four forms as a function of simulation time. (**A**) WT system; (**B**) C137SSH system; (**C**) 2CSSH system; (**D**) 2CSSH-pS267 system.

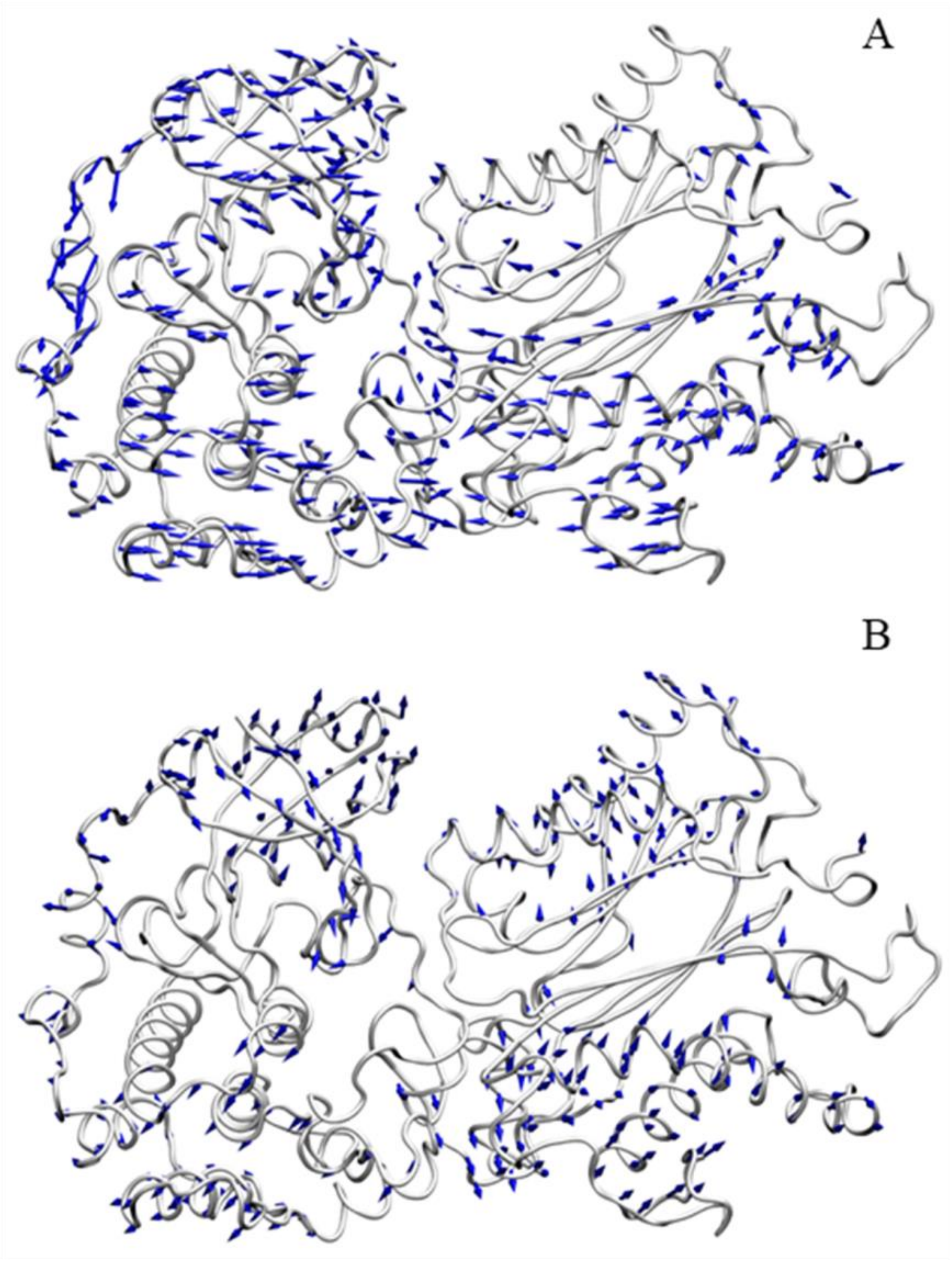

Figure S2. Collective motions in SnRK2.6–HAB1 simulations. Porcupine representation of PC1 (**A**) and PC2 (**B**). The blue spikes show the direction and relative amplitude of motion of each residue along the PC.

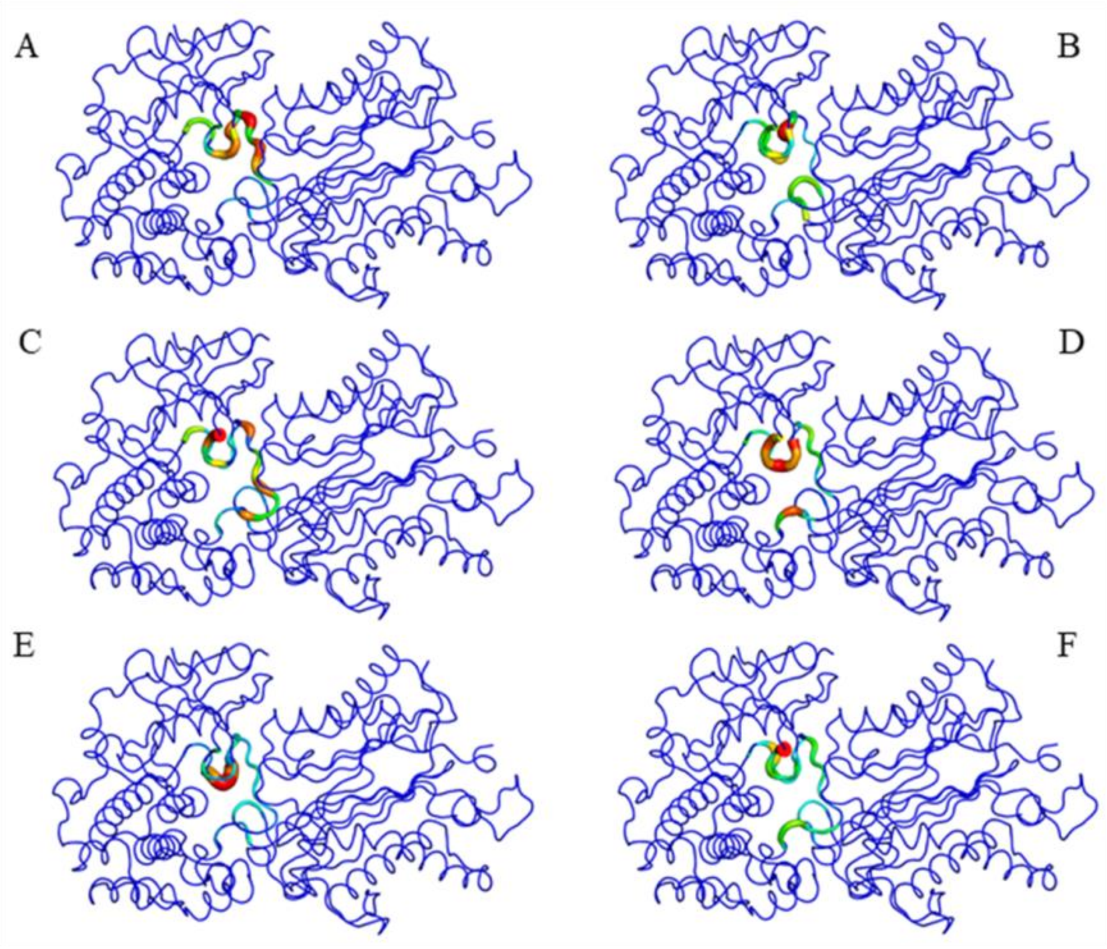

Figure S3. Mapping of Cα RMS fluctuations of the first 2 eigenmodes for the loops (P-loop, A-loop, and flap loop) onto SnRK2.6–HAB1 structure. The width and color (from blue to red) of sausage presentation related to the RMS fluctuation values. The left panels are corresponding to the first mode, and the right corresponding to the second mode. (**A**,**B**) WT system; (**C**,**D**) 2CSSH system; (**E**,**F**) 2CSSH-pS267 system.

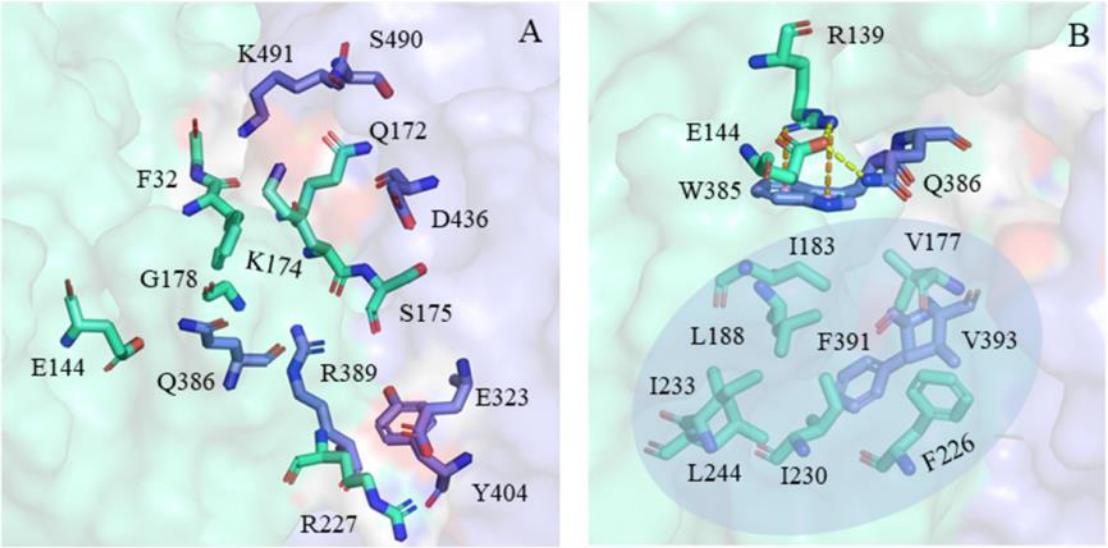

Figure S4. The residues participated in SnRK2.6–HAB1 association. (**A**) Interfacial residues. The ones in SnRK2.6 colored in limegreen and the ones in HAB1 colored in slate, all shown in sticks. (**B**) The cation-pi interaction between R139 in SnRK2.6 and W385 in HAB1. The center of mass of the sidechain rings of W385 is shown in magenta sphere. The hydrogen-bond interaction between E144 in SnRK2.6 and Q386 in HAB1. Hydrophobic residues form a hydrophobic core in zone of binding, represented by oval cycle.

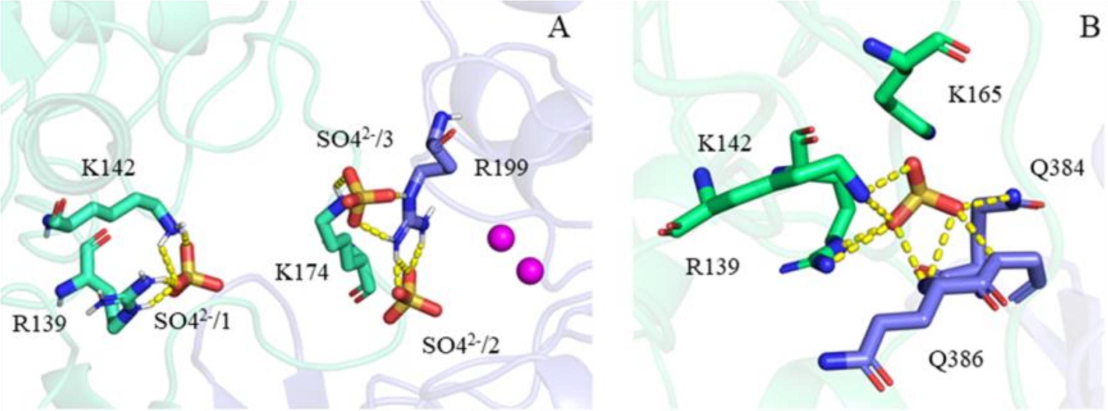

Figure S5. Structural presentation of sulfate ions resolved in the crystal structure of SnRK2.6–HAB1 complex. (**A**) Three sulfate ions in SnRK2.6–HAB1 interface and several important residues coordinating with SO_4^{2-} are shown as sticks. (**B**) Enlarged view of SO_4^{2-}/1.

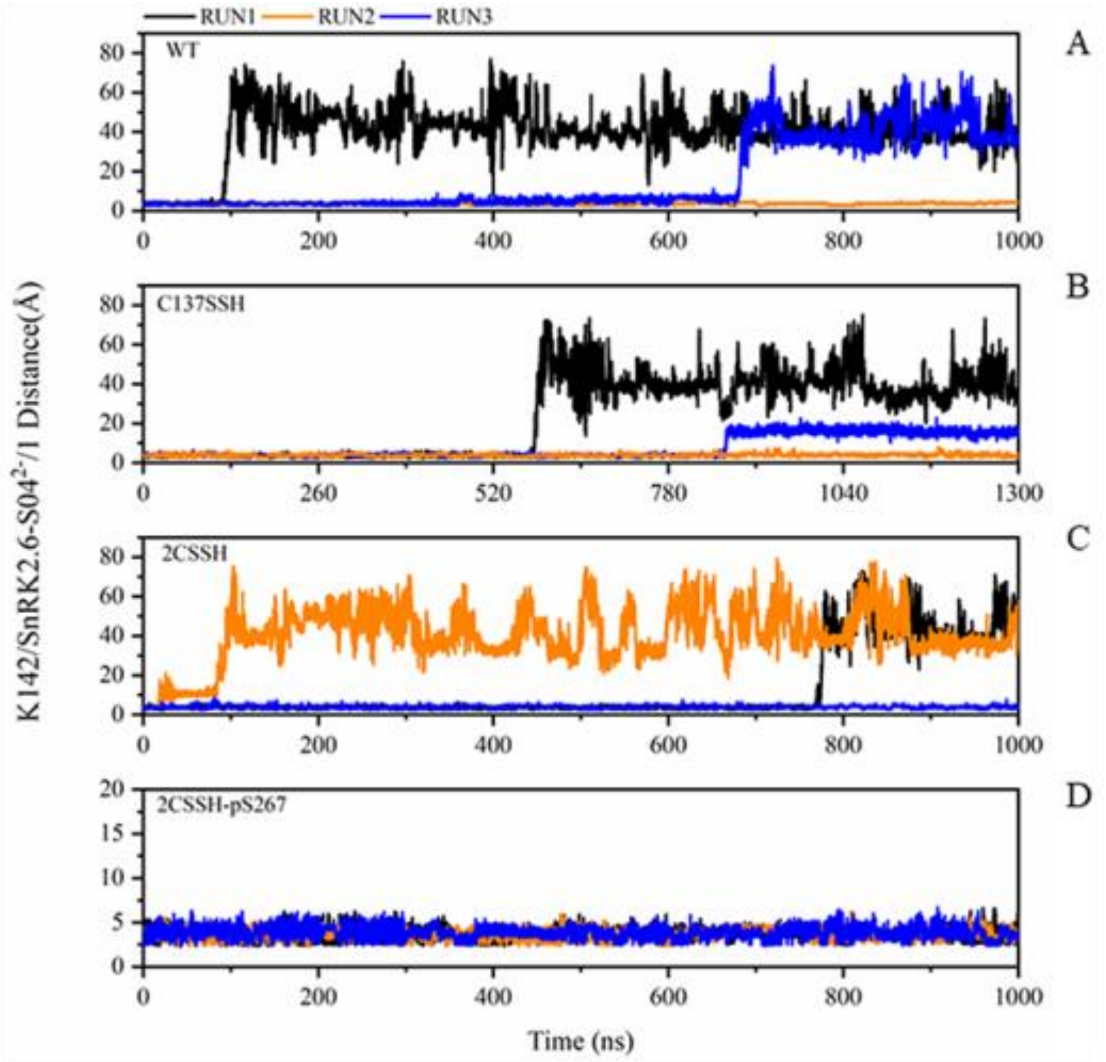

Figure S6. The distance between SO4^{2-}/1 and residue K142 in SnRK2.6 as a function of simulation time. (**A**) WT system; (**B**) C137SSH system; (**C**) 2CSSH system; (**D**) 2CSSH-pS267 system.

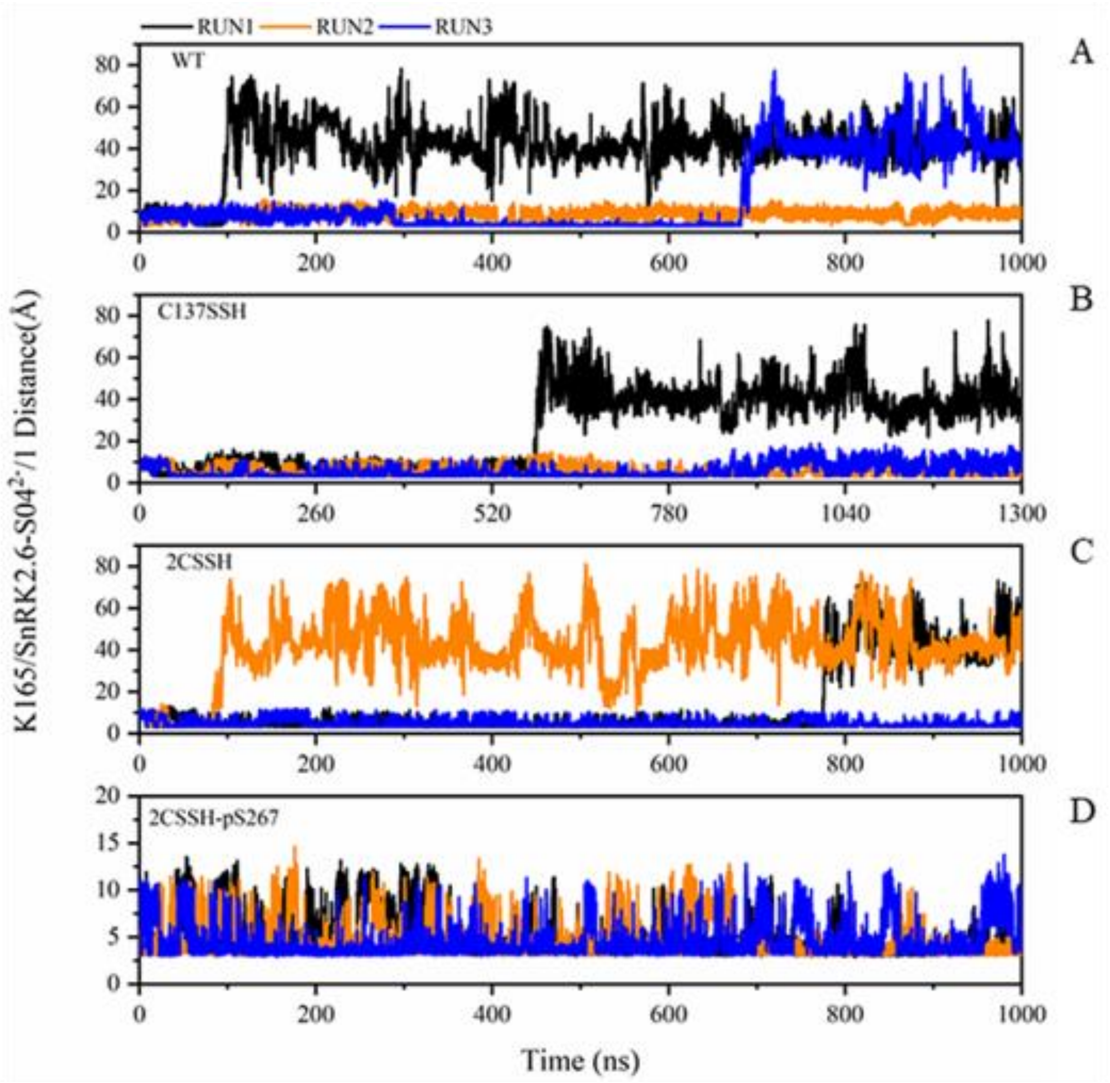

Figure S7. The distance between SO4^{2-}/1 and residue K165 in SnRK2.6 as a function of simulation time. (**A**) WT system; (**B**) C137SSH system; (**C**) 2CSSH system; (**D**) 2CSSH-pS267 system.
